# Supplementary material for: Time-series transcriptome analysis identified differentially expressed genes in broiler chicken infected with mixed Eimeria species
Source: Front Genet. 2022 Aug 8;13:886781. doi: 10.3389/fgene.2022.886781 (PMC9393255; doi:10.3389/fgene.2022.886781)
Supplement: Supplementary file 2 [file DataSheet1.ZIP › 7dpi_GO.Gsea.1625106495923/GOCC_LARGE_RIBOSOMAL_SUBUNIT.html]

Details for gene set GOCC\_LARGE\_RIBOSOMAL\_SUBUNIT[GSEA]

|  || Dataset | TMM\_7dpi\_gct\_format\_7dpi\_gct\_format.Class\_4dpi.cls #PC\_versus\_NC.Class\_4dpi.cls #PC\_versus\_NC\_repos |
| Phenotype | Class\_4dpi.cls#PC\_versus\_NC\_repos |
| Upregulated in class | 1 |
| GeneSet | GOCC\_LARGE\_RIBOSOMAL\_SUBUNIT |
| Enrichment Score (ES) | 0.6226486 |
| Normalized Enrichment Score (NES) | 3.09191 |
| Nominal p-value | 0.0 |
| FDR q-value | 0.0 |
| FWER p-Value | 0.0 |
Table: GSEA Results Summary

  

Fig 1: Enrichment plot: GOCC\_LARGE\_RIBOSOMAL\_SUBUNIT      
 Profile of the Running ES Score & Positions of GeneSet Members on the Rank Ordered List

  

| SYMBOL | TITLE | RANK IN GENE LIST | RANK METRIC SCORE | RUNNING ES | CORE ENRICHMENT || 1 | MRPL30 | na | 351 | 0.883 | 0.0025 | Yes |
| 2 | NDUFAB1 | na | 356 | 0.876 | 0.0339 | Yes |
| 3 | MRPL43 | na | 504 | 0.741 | 0.0484 | Yes |
| 4 | MRPL23 | na | 564 | 0.704 | 0.0690 | Yes |
| 5 | MRPL12 | na | 579 | 0.694 | 0.0930 | Yes |
| 6 | MRPL57 | na | 643 | 0.665 | 0.1118 | Yes |
| 7 | MPV17L2 | na | 697 | 0.638 | 0.1304 | Yes |
| 8 | MRPL58 | na | 755 | 0.609 | 0.1477 | Yes |
| 9 | MRPL54 | na | 779 | 0.596 | 0.1674 | Yes |
| 10 | MRPL35 | na | 853 | 0.571 | 0.1820 | Yes |
| 11 | MRPL10 | na | 872 | 0.563 | 0.2009 | Yes |
| 12 | MRPL55 | na | 880 | 0.561 | 0.2206 | Yes |
| 13 | RPL26L1 | na | 1025 | 0.512 | 0.2271 | Yes |
| 14 | MRPL21 | na | 1141 | 0.472 | 0.2345 | Yes |
| 15 | MRPL15 | na | 1217 | 0.450 | 0.2445 | Yes |
| 16 | MRPL40 | na | 1282 | 0.431 | 0.2548 | Yes |
| 17 | MRPL45 | na | 1312 | 0.427 | 0.2678 | Yes |
| 18 | MRPL46 | na | 1380 | 0.409 | 0.2770 | Yes |
| 19 | MRPL28 | na | 1382 | 0.409 | 0.2918 | Yes |
| 20 | NSUN4 | na | 1403 | 0.404 | 0.3047 | Yes |
| 21 | MRPL9 | na | 1437 | 0.396 | 0.3163 | Yes |
| 22 | MRPL3 | na | 1475 | 0.387 | 0.3272 | Yes |
| 23 | MRPL42 | na | 1521 | 0.375 | 0.3370 | Yes |
| 24 | MRPL19 | na | 1535 | 0.370 | 0.3493 | Yes |
| 25 | MRPL47 | na | 1550 | 0.367 | 0.3614 | Yes |
| 26 | RPL38 | na | 1576 | 0.361 | 0.3724 | Yes |
| 27 | RPL36 | na | 1577 | 0.361 | 0.3855 | Yes |
| 28 | MRPL1 | na | 1639 | 0.348 | 0.3930 | Yes |
| 29 | MRPL34 | na | 1672 | 0.339 | 0.4026 | Yes |
| 30 | MRPL33 | na | 1696 | 0.333 | 0.4127 | Yes |
| 31 | MRPL37 | na | 1736 | 0.324 | 0.4212 | Yes |
| 32 | RPL23A | na | 1779 | 0.313 | 0.4290 | Yes |
| 33 | RPL29 | na | 1780 | 0.313 | 0.4403 | Yes |
| 34 | MRPL44 | na | 1799 | 0.307 | 0.4499 | Yes |
| 35 | RPLP1 | na | 1813 | 0.304 | 0.4598 | Yes |
| 36 | RPL36A | na | 1833 | 0.301 | 0.4691 | Yes |
| 37 | RPL19 | na | 1834 | 0.300 | 0.4800 | Yes |
| 38 | MRPL2 | na | 1843 | 0.299 | 0.4902 | Yes |
| 39 | MRPL16 | na | 1884 | 0.291 | 0.4973 | Yes |
| 40 | RPL39L | na | 1966 | 0.277 | 0.5006 | Yes |
| 41 | RPL37A | na | 1983 | 0.274 | 0.5091 | Yes |
| 42 | MRPL53 | na | 1984 | 0.273 | 0.5190 | Yes |
| 43 | MRPL41 | na | 2082 | 0.257 | 0.5202 | Yes |
| 44 | MRPL24 | na | 2093 | 0.254 | 0.5286 | Yes |
| 45 | MRPL13 | na | 2109 | 0.251 | 0.5364 | Yes |
| 46 | RPL22 | na | 2201 | 0.237 | 0.5374 | Yes |
| 47 | MRPL48 | na | 2228 | 0.232 | 0.5436 | Yes |
| 48 | RPL35A | na | 2241 | 0.231 | 0.5509 | Yes |
| 49 | RSL24D1 | na | 2281 | 0.225 | 0.5558 | Yes |
| 50 | MRPL14 | na | 2284 | 0.224 | 0.5638 | Yes |
| 51 | RPL34 | na | 2286 | 0.224 | 0.5718 | Yes |
| 52 | RPL27 | na | 2300 | 0.222 | 0.5788 | Yes |
| 53 | RPL14 | na | 2343 | 0.215 | 0.5830 | Yes |
| 54 | MRPS18A | na | 2363 | 0.213 | 0.5891 | Yes |
| 55 | RPL23 | na | 2383 | 0.209 | 0.5951 | Yes |
| 56 | RPL7L1 | na | 2403 | 0.207 | 0.6010 | Yes |
| 57 | MRPL18 | na | 2512 | 0.189 | 0.5988 | Yes |
| 58 | RPL7 | na | 2670 | 0.168 | 0.5917 | Yes |
| 59 | MRPL39 | na | 2682 | 0.166 | 0.5968 | Yes |
| 60 | RPL31 | na | 2693 | 0.164 | 0.6019 | Yes |
| 61 | RPL24 | na | 2734 | 0.160 | 0.6044 | Yes |
| 62 | MRPL22 | na | 2747 | 0.158 | 0.6091 | Yes |
| 63 | MRPL20 | na | 2783 | 0.153 | 0.6116 | Yes |
| 64 | RPL21 | na | 2810 | 0.149 | 0.6149 | Yes |
| 65 | RPL32 | na | 2870 | 0.142 | 0.6151 | Yes |
| 66 | RPL13 | na | 2902 | 0.138 | 0.6175 | Yes |
| 67 | MRPL38 | na | 2918 | 0.137 | 0.6212 | Yes |
| 68 | RPLP2 | na | 3040 | 0.121 | 0.6154 | Yes |
| 69 | RPL6 | na | 3048 | 0.121 | 0.6192 | Yes |
| 70 | RPL27A | na | 3078 | 0.116 | 0.6209 | Yes |
| 71 | RPL9 | na | 3146 | 0.107 | 0.6192 | Yes |
| 72 | MRPL27 | na | 3152 | 0.107 | 0.6226 | Yes |
| 73 | MRPL51 | na | 3225 | 0.098 | 0.6202 | No |
| 74 | RPL30 | na | 3365 | 0.078 | 0.6113 | No |
| 75 | RPL11 | na | 3414 | 0.072 | 0.6099 | No |
| 76 | RPL7A | na | 3462 | 0.067 | 0.6084 | No |
| 77 | RPL35 | na | 3487 | 0.064 | 0.6087 | No |
| 78 | RPL18A | na | 3668 | 0.045 | 0.5952 | No |
| 79 | MRPL32 | na | 3822 | 0.025 | 0.5833 | No |
| 80 | RPL10A | na | 3828 | 0.024 | 0.5838 | No |
| 81 | RPL3 | na | 3883 | 0.018 | 0.5799 | No |
| 82 | RPL37 | na | 4179 | -0.015 | 0.5557 | No |
| 83 | RPL8 | na | 4274 | -0.027 | 0.5487 | No |
| 84 | RPL5 | na | 4509 | -0.051 | 0.5309 | No |
| 85 | MALSU1 | na | 4809 | -0.082 | 0.5088 | No |
| 86 | RPL15 | na | 4832 | -0.084 | 0.5100 | No |
| 87 | RPL12 | na | 4912 | -0.094 | 0.5068 | No |
| 88 | RPL4 | na | 5112 | -0.114 | 0.4942 | No |
| 89 | RPLP0 | na | 5368 | -0.137 | 0.4778 | No |
| 90 | RBM3 | na | 5497 | -0.149 | 0.4724 | No |
| 91 | MRPL17 | na | 5768 | -0.177 | 0.4562 | No |
| 92 | MRPL50 | na | 6658 | -0.271 | 0.3914 | No |
| 93 | RPL17 | na | 7417 | -0.353 | 0.3406 | No |
| 94 | MRPS30 | na | 8183 | -0.444 | 0.2924 | No |
| 95 | NSUN3 | na | 10405 | -0.791 | 0.1347 | No |
Table: GSEA details [plain text format]

  

Fig 2: GOCC\_LARGE\_RIBOSOMAL\_SUBUNIT      
 Blue-Pink O' Gram in the Space of the Analyzed GeneSet

  

Fig 3: GOCC\_LARGE\_RIBOSOMAL\_SUBUNIT: Random ES distribution      
 Gene set null distribution of ES for **GOCC\_LARGE\_RIBOSOMAL\_SUBUNIT**
